# Agreement Between Panoramic and Lateral Cephalometric Radiographs for Measuring the Gonial Angle

**DOI:** 10.5812/iranjradiol.8444

**Published:** 2012-11-20

**Authors:** Maryam Zangouei-Booshehri, Hossein-Agha Aghili, Mojtaba Abasi, Fatemeh Ezoddini-Ardakani

**Affiliations:** 1 Department of Oral and Maxillofacial Radiology, Faculty of Dentistry, Shahid Sadoughi University of Medical Sciences, Yazd, Iran; 2 Department of Orthodontics, Faculty of Dentistry, Shahid Sadoughi University of Medical Sciences, Yazd, Iran; 3 Faculty of Dentistry, Shahid Sadoughi University of Medical Sciences, Yazd, Iran

**Keywords:** Radiography, Panoramic, Cephalometry, Orthognathic Surgery, Gonial Angle

## Abstract

**Background:**

The gonial angle is one of the most important measurements required for orthodontic treatment and orthognathic surgery. It is difficult to determine the accurate measurement of each gonial angle on cephalometric radiographs because of superimposition of the left and right angles.

**Objectives:**

The aim of the present study was to determine the right and left gonial angles on panoramic radiographs and to compare them with an evaluated cephalometric sample.

**Patients and Methods:**

A total of 80 panoramic and 80 cephalometric radiographs were obtained from 6 to 12-year-old children and the gonial angle was determined by the tangent of the inferior border of the mandible and the most distal aspect of the ascending ramus and the condyleon both panoramic and cephalometric radiographs. We used Pearson’s correlation coefficient and paired t-test for comparison.

**Results:**

The mean gonial angle was 127.07 ± 6.10 and 127.5 ± 6.67 degrees on panoramic and cephalometric radiographs, respectively. There was no statistically significant difference between the measured gonial angles on panoramic and cephalometric radiographs and also no difference between the right and left (both Ps = 0.18)

**Conclusion:**

The value of the gonial angle measured on panoramic radiography was the same as that measured on the routinely used cephalometric radiography.

## 1. Background

Treatment of mandibular teeth anomalies either by orthodontic methods or by interventional surgery requires paying attention to special conditions. In order to plan an orthodontic treatment method, some documents such as general observation, clinical examination, study moulds and related radiographs are available (1). Panoramic radiography is frequently used in orthodontic practice to provide important information about the teeth, their axial inclinations, maturation periods and surrounding tissues (2-5). Currently, lateral and anteroposterior projections are used for cephalometry. However, in this method, measuring individual gonial angles is difficult, because lateral cephalograms are superimposed by other images. This disadvantage is not encountered in panoramic imaging which is being used increasingly for jaw examination (1). Others stated that there was a great individual variation in gonial angle distortion and showed that the gonial angle differs by age and different types of malocclusion (6-9). One of the most important angles for determining orthodontic or surgical treatment plans is the gonial angle, which is usually extracted from lateral cephalometric radiographs (1).

Some studies have shown that the left and right gonial angles can be separately measured by panoramic radiography which is a simple and repeatable radiographic method. Studies have shown that the difference in the measured gonial angle in two consecutive panoramic radiographs is only one degree (10, 11). Since the gonial angle can be determined more easily in an orthopantomogram (OPG) than in a lateral cephalogram, we decided to compare the efficiency of panoramic and lateral cephalometric radiographs in the measurement of gonial angles.

## 2. Objectives

The aim of this study was to assess the accuracy of panoramic imaging in measuring the right and left gonial angles by comparison with evaluated cephalometric samples.

## 3. Patients and Methods

In this cross-sectional study, we assessed the agreement between gonial angles measured in panoramic and lateral cephalometric radiographs. Eighty panoramic and 80 lateral cephalometric radiographs were obtained from 6 to 12-year-old children referred to the orthodontic department of the dental faculty. The subjects with a previous history of facial or mandibular surgery or syndromes affecting the jaw or face were excluded from the study. The quality of radiographs was checked according to radiographic standards and radiographs with technical, exposure or fixation faults were excluded from the study. All radiographs were obtained at the Oral and Maxillofacial Radiology department of Yazd Dental Faculty. Panoramic and cephalometric images were acquired with a Planmeca 2002 EC proline multitomographic X-ray unit (Planmeca Co., Helsinki, Finland). They were obtained with a constant 12 mA, 80 KVP and 18 s exposure through 2.5 mm Al filtration. Regular Kodak Lanex (Eastman Kodak Co, Rochester, NY) intensifying screens (15 × 30 cm and 18 × 24 cm cassette) were used in this study. Films were developed in an automatic film processor (Velopex, Extra-X, Medivance Instruments Ltd, London, UK) with standard solutions. The total time of processing was 4 minutes at 27 ◦C working temperature.

**Figure 1:**
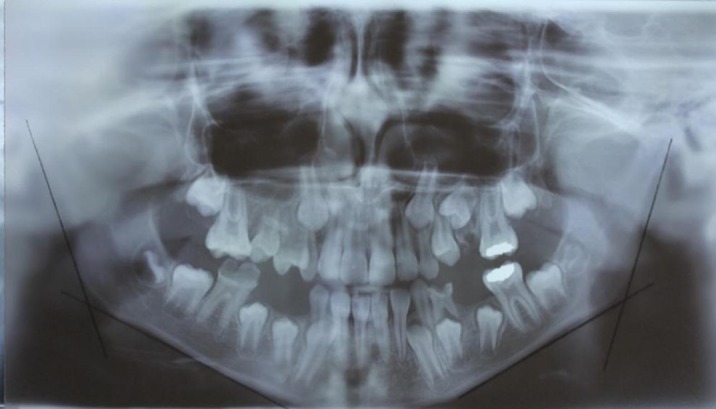
The gonial angle in panoramic radiography

**Figure 2:**
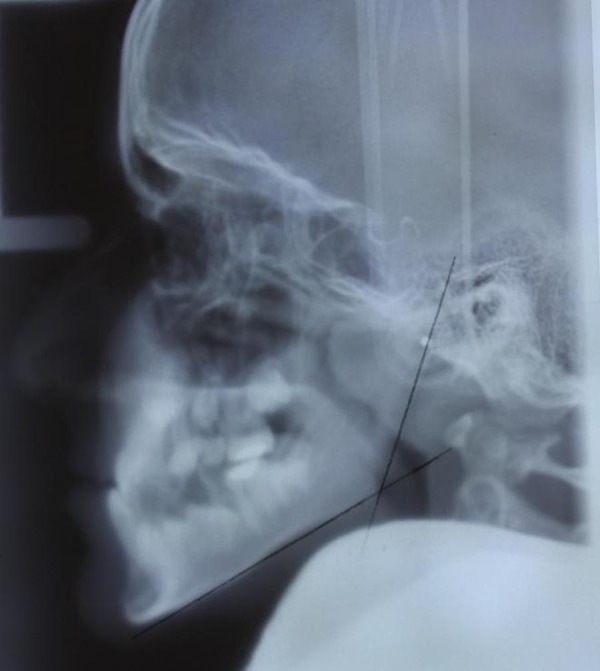
The gonial angle in lateral cephalometry

The radiographs were viewed and evaluated by two expert radiologists. The gonial angle in the intersection of the ramal plane (Ar-Go) and mandibular plane (Go-Gn) was traced on paper and measured using a protractor with 1 degree accuracy (Figures 1 and 2). The measurements in panoramic and cephalometric radiographs were performed by an expert oral and maxillofacial radiologist. The type of occlusion was determined based on angle classification, the diagnosis recorded in the patient’s medical file and the chronological age. The data were analyzed by SPSS 17 for Windows (SPSS Inc., Chicago, Illinois, USA) using Pearson’s correlation coefficient and paired samples t test.

## 4. Results

**Figure 3:**
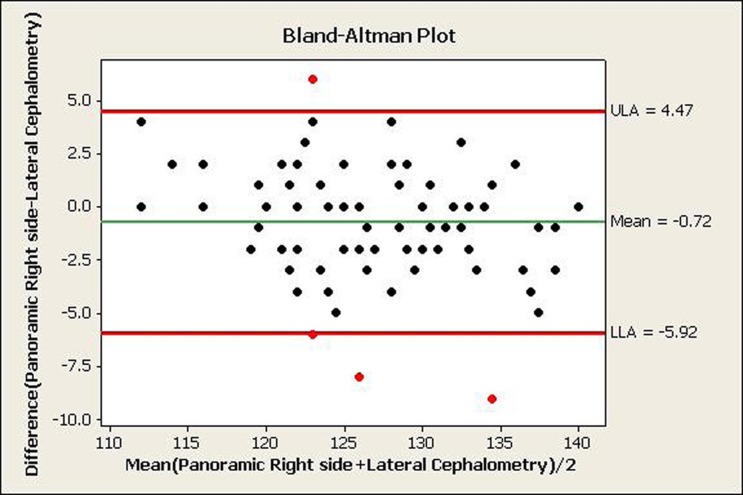
The agreement between right panoramic and lateral cephalometric radiographs

**Figure 4:**
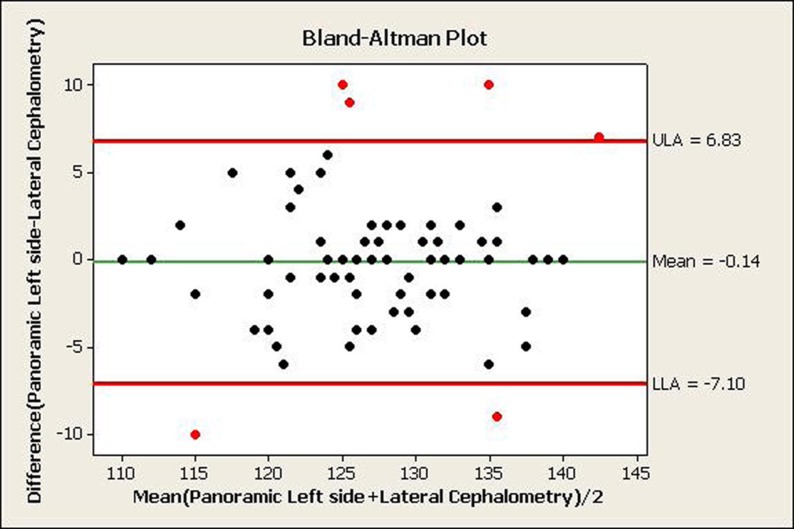
The agreement between left panoramic and lateral cephalometric radiographs

A total of 80 panoramic and 80 cephalometric radiographs were obtained from 6 to 12-year-old children and the gonial angle was determined by the tangent of the lower border of the mandible and the distal border of the ascending ramus and the condyle on both panoramic and cephalometric radiographs. Table 1 shows the mean gonial angle measured by the two methods. Table 2, Figures 3 and 4 show the agreement between the two methods (i.e. left and right panoramic and cephalometric radiographs) for measuring the gonial angles by Pearson’s correlation coefficient and Bland-Altman plot. This plot shows the mean of differences between the two measurement methods with a 95% confidence interval. The agreement between the two methods is observed when most of the dots are located in this area. Therefore, Figures 3 and 4 show acceptable agreement between the two methods.

**Table 1:** Mean and Standard Deviation of Gonial Angle on Panoramic and Cephalometric Radiographs (Degrees)

Variables	Mean	SD
** OPG L^a^**	126.77	6.10
** OPG R**	127.36	6.88
** OPG**	127.07	6.10
** Cephalometry**	127.5	6.67

^a^Panoramic Radiography

**Table 2:** Correlation Between Right and Left Panoramic and Cephalometric Radiographs for Measurement of the Gonial Angle

Variables	Pearson’s Correlation Coefficient	*P* value
** Left and right panoramic gonial angles**	0.82	0.01
** Left panoramic and cephalometric gonial angles**	0.918	0.01
** Right panoramic and cephalometric gonial angles**	0.863	0.01

Evaluation of the mean and standard deviation of the gonial angle on panoramic and cephalometric radiographs showed that the angle was not significantly different between the two genders. Tables 3, 4 and 5 compare the mean and standard deviation of the gonial angle measured by two different methods in different age groups, different genders and different types of occlusion.

**Table 3:** Mean and Standard Deviation of the Gonial Angle in Panoramic and Cephalometric Radiographs (Degrees) in Different Age Groups

	Left Panoramic Gonial Angle		Right Panoramic Gonial Angle		Cephalometric Gonial Angle	
	Mean	SD	Mean	SD	Mean	SD
**Below 9 years**	126.82	6.57	127.19	6.95	128.15	7.33
**9-12 years**	126.63	5.7	127.52	6.97	126.79	6.03
***P *value**	0.883		0.887		0.347	

**Table 4:** Mean and Standard Deviation of the Gonial Angle in Panoramic and Cephalometric Radiographs (Degrees) in Different Genders

Gender	Left Panoramic Gonial Angle		Right Panoramic Gonial Angle		Cephalometric Gonial Angle	
	Mean	SD	Mean	SD	Mean	SD
**Male**	127.54	6.95	127.53	7.91	127.72	7.55
**Female**	126.4	5.68	127.27	6.40	127.38	6.29
***P* value**	0.441		0.875		0.832	

**Table 5:** Mean and Standard Deviation of the Gonial Angle in Panoramic and Cephalometric Radiographs (Degrees) in Different Types of Occlusion

Type of Occlussion	Left Panoramic Gonial Angle		Right Panoramic Gonial Angle		Cephalometric Gonial Angle	
	Mean	SD	Mean	SD	Mean	SD
**I**	127.13	6.48	127.93	7.6	128.16	7.20
**II**	126.32	5.47	127.23	6.33	127.17	5.74
**III**	127.55	7.84	126	7.19	126.67	9.11
**P value**	0.795		0.752		0.769	

## 5. Discussion

The gonial angle is also the representation of the form of the mandible. This angle has an important role in predicting growth and it also has specific effects initially on growth, profile changes and the condition of the anterior teeth of the lower jaw (1).

This study was performed to assess and compare the measurement of the gonial angle from panoramic radiographs and lateral cephalograms in children aged 6-12 years. The use of panoramic radiography in the determination of the gonial angle has been studied and the results have shown that gonial angle measurement on the panoramic radiograph is an accurate and repeatable criterion (10, 12). The variation in the angle measured by this method considering the usual and acceptable errors is 0.5-1 degree (13) and the variation on cephalometric view is 2.2-3.6 degrees (2).

Assessment of the angle on the right and left panoramic radiographs makes it possible to accurately evaluate the changes after orthodontic treatment (13). Panoramic radiography has been reported to have potential in measuring mandibular inclination and gonial angle. It has been successfully used for determining gonial angle which is a good indicator of mandibular steepness and growth direction. As OPG is routinely requested by dentists during dental examination, it seems to be a useful feature of this modality for determining growth direction, so that dentists can detect vertical growth problems.

The mean value of gonial angle of the subjects in the present study in OPG and cephalometry was 127.07 ± 6.10 and 127.5 ± 6.67 degrees, respectively in comparison to a study performed by Updegrave et al. in which these angles were 127.3 ± 6.5 and 122.4 ± 6.6 degrees respectively, that may be due to the genetic differences in growth patterns (14). Matilla (1) reported that the accuracy of the measurement of the gonial angle on panoramic radiograph is similar to cephalometry, although in a study reported by Akcam et al., regression analysis showed that the forecasting capability of vertical measurements on panoramic radiographs is 11-20% of cephalometry (5). He stated that even though panoramic radiographs provide information on the vertical dimensions of craniofacial structures, clinicians should be vigilant when predicting skeletal cephalometric parameters from panoramic radiographs because of their lower predictability. This is contrary to what other researchers believe (5). The authors did not observe any difference between the right and left gonial angles. Similar results have been stated in various studies (13, 15, 16), though the measurement of the gonial angle on the panoramic radiograph is highly affected by the head position and the usual panoramic malformation can affect the angle measurement (14). So we standard the panoramic technique by maximizing the accuracy of the head position using indicating lights, bite grove biting in the correct place (correct focal trough selection) and the selection of the shape of the mandible in the imaging program. In addition to use the intersection of the ramal plan and mandibular plan as the gonial angle, there is no chance for false localization of the gonion point (GO) (15).

In our study, there was no significant difference between the mean gonial angles in different genders, age groups or types of malocclusions from the two different types of radiographs, which was in agreement with the results reported by Altonen (7). Raustia et al. claimed that gender had insignificant impact on the size of the gonial angle (16). Some previous studies have reported a difference in gonial angle between the two genders (15-17). Gungor et al. showed a difference in the left gonial angle between the two genders (11). Larheim and Svanaes also stated that both panoramic radiographs and lateral cephalograms were accurate in determining the gonial angle and there was no significant difference between the right and left sides in panoramic radiography (18). Many other studies have confirmed the usefulness of panoramic radiography for measuring the gonial angle; the left and right gonial angles do not overlap on panoramic radiography contrary to cephalometry (15-17, 19). However, Nohadani et al. compared longitudinal vertical facial and dentoalveolar changes using panoramic radiographs with measurements on lateral cephalometric radiographs. They reported that panoramic dental radiographs are not useful for evaluating vertical facial parameter changes during time (20), but as confirmed previously, angular measurements in panoramic images are more reliable than vertical measurements because they are not influenced by the image distortion, especially in posterior and lateral aspects of the mandible. So angular distortion in panoramic images is within the acceptable range and they could be used for clinical measurements if the images are prepared perfectly and without technical errors (21). There is no data about the effect of occlusion groups on the gonial angle and in the present study, there was no significant difference between different occlusion types. In the prediction of the treatment prognosis, concluding that in the evaluation of growth parameters in which subtle changes are very important, PRs are not suitable for analyzing longitudinal changes in vertical facial and dentoalveolar parameters. In addition, left and right gonial angles can be determined using an OPG as there is no interferences caused by superimposed images. So for measuring gonial angle, an OPG seems to be a better choice than lateral cephalogram. Huumonen et al. assessed the gonial angle of the mandible on the panoramic radiographs. They reported that the morphology of the mandible changes as a consequence of tooth loss, which can be expressed as a widening of the gonial angle and shortening of the ramus and condylar height (22). We did not encounter with this point because of the samples age range.

It may be concluded that panoramic radiography can be used to determine the gonial angleas accuratelyasa lateral cephalogram. Furthermore, in panoramic radiography the right and left gonialangle scan is measured easily without superim position of anatomic landmarks, which occurs frequently in a lateral cephalogram. Therefore, it seems that panoramic radiography which is a simple, inexpensive and available radiologic technique can be used for determination of the left and right gonial angles.
